# Is MR Imaging of the Cervical Spinal Cord Sufficient for Patients with Suspected Multiple Sclerosis?

**DOI:** 10.1007/s00062-025-01613-5

**Published:** 2026-01-21

**Authors:** Isabelle Riederer, Matthias Bussas, Markus Lauerer, Laura Harabacz, Viktor Pineker, Malek El Husseini, Nico Sollmann, Claus Zimmer, Jan S. Kirschke, Mark Mühlau

**Affiliations:** 1 Institute for Neuroradiology, TUM University Hospital, School of Medicine and Health, Technical University of Munich (TUM), Munich, Germany; 2 Department of Neurology, TUM University Hospital, School of Medicine and Health, Technical University of Munich (TUM), Munich, Germany; 3 TUM-NIC, TUM Neuroimaging Center, TUM University Hospital, School of Medicine and Health, Technical University of Munich (TUM), Munich, Germany; 4 Department of Diagnostic and Interventional Radiology, University Hospital Ulm, Ulm, Germany

**Keywords:** Multiple sclerosis, Magnetic resonance imaging, Spinal cord

## Abstract

**Purpose:**

Lesions in the spinal cord (SC) can be found in up to 83% of patients with multiple sclerosis (MS). As they are mainly located in the cervical segment, many centers exclude the thoracic part from SC imaging. The aim of our study was to quantify the decrease in sensitivity resulting from this approach.

**Methods:**

MR images (3T) of 543 consecutive patients with clinically isolated syndrome (CIS) (*n*: 37) and MS (*n*: 506) were analyzed retrospectively. Lesions were segmented semi-automatically on axial T2-weighted images of the whole SC using BrainSeg3D. The volume of lesions was related to vertebral levels.

**Results:**

Altogether 1782 lesions (CIS: 19; MS: 1763) were found in 409 patients. 70% of the lesion volume was located in the SC above the 3rd thoracic vertebral body, in a segment that is commonly covered by an isolated examination of the cervical SC. However, 26 patients (6%) showed lesions exclusively below the 3rd thoracic vertebral body, thus 94% of all patients with SC lesions could be detected with isolated MR imaging of the cervical SC.

**Conclusion:**

Though the majority of lesions can be detected in an isolated examination of the upper part of the SC, some patients showed lesions exclusively below the 3rd thoracic vertebral body. We recommend routine scanning of the whole SC in suspected MS.

## Introduction

Multiple sclerosis (MS) is a chronic autoimmune inflammatory disease that affects both the brain and the spinal cord (SC). SC lesions are found in up to 83% of patients with MS [1]. The recently revised MAGNIMS-CMSC-NAIMS consensus guidelines [2] emphasize the importance of examining the SC. The involvement of the SC fulfills one of the specific McDonald criteria [3] for the dissemination in space in addition to intra-/juxtacortical, periventricular, infratentorial localization and, as recently added, the optic nerve on brain magnetic resonance imaging (MRI). Inclusion of the SC in these criteria increases the likelihood of dissemination in space from 66% to 85% [1]. Furthermore, lesions in the SC may help to exclude differential diagnoses as they are less common in other neurological diseases and do not occur in healthy ageing [4]. In addition MRI of the SC may help to differentiate MS from neuromyelitis optica spectrum disorders (NMOSD) and myelon oligodendrocyte glycoprotein antibody (MOG-AD) as the distribution, localization and morphology of the lesions are pathognomonic. Lesion extension or SC atrophy of at least three contiguous segments are specific for NMOSD. The involvement of conus or thoracolumbar cord is an indication of MOG-AB [5].

However, MRI of the SC remains technically challenging due to its thin and long structure and artifacts caused by pulsation or respiration. Due to the long structure of the SC, at least 2–3 stacks are necessary to cover the whole SC with MRI. Recommendations for MRI protocols suggest at least two out of five complementary sagittal sequences for MRI of the SC (T2-, PD-TSE, T2-STIR, PSIR and MP[2]RAGE) supplemented by axial T2-weighted sequences to differentiate between true lesions and artifacts, whereby it must be pointed out, that the data (sagittal and axial T2w MRI) were not recorded fully 2021/2024 MAGNIMS-CMSC-NAIMS consensus compliant because of the gap [2]. However, a detailed MRI protocol is time-consuming and may not be well tolerated by some patients. As most of the lesions (56%) are located in the cervical segment of the SC [1], many centers, therefore, exclude the thoracic part of the SC in MRI [6–10].

Therefore, the aim of our study was to quantify the decrease in sensitivity resulting from incomplete SC coverage in MRI scans of patients with suspected MS.

## Methods

### Patients

This study was performed retrospectively as part of the single-centre cohort study on MS at the Technical University of Munich (TUM-MS). The study was approved by the internal review board and conducted in accordance with the Declaration of Helsinki. Patients had given written informed consent for the use of their clinical and paraclinical data for research purposes. Inclusion criteria were a diagnosis of clinically isolated syndrome (CIS) or MS and an age between 18 and 60 years. To achieve a uniform classification of patients, all patients were reclassified according to the 2017 diagnostic criteria [11] with full access to the neurologist’s medical records. Patients with CIS were defined by a first clinical event suggestive of a relapsing-remitting MS (RRMS) and meeting the criteria for dissemination in space but not for dissemination in time. MS patients included those with a relapsing-remitting course (RRMS) and those with a primary progressive (PPMS) or secondary progressive (SPMS) course.

### MRI Acquisition and Analysis

Data were analyzed retrospectively, and MRI of the SC were acquired between January 2009 and June 2018 during routine clinical practice. Imaging was performed on 3‑Tesla scanners (Achieva dStream, Ingenia, or Ingenia Elition from Philips Healthcare and Magnetom Verio from Siemens Healthineers) using an anterior body coil. All scans included 2D T2-weighted (w) turbo spin echo (TSE) sequences in sagittal and axial orientation with a slice thickness of 2 mm and a gap of 0.2 mm (sagittal) or a slice thickness of 4 mm and a gap of 1 mm (axial). The typical field of view (FOV) of axial scans was 115 mm with an in-plane spatial resolution of 0.4 mm (ranging from 0.3 to 0.5 mm) and were acquired in three consecutive stacks. In axial acquisitions, mean TE was approximately 99 ms and mean TR approximately 5400 ms. Echo train lengths were around 19–21, and compressed SENSE (factor ~1.7) was again applied on Philips scanners only. Sagittal scans had a typical FOV of 250 mm with an in-plane spatial resolution of 0.9 mm (ranging from 0.8 to 1 mm) and were acquired in two consecutive stacks. In sagittal acquisitions, average TE was approximately 111 ms and average TR 3000 ms; echo train lengths ranged from 21 to 30, and parallel imaging (compressed SENSE) was used on the Philips systems (factor ~1.7) but not on the Siemens scanner.

All scans were converted to the NIFTI file format and segmented using the BrainSeg3D software, version 2.2.1 (http://lit.fe.uni-lj.si/tools.php?lang=eng). First, we segmented the SC area in axial scans using a region-growing algorithm that had to be initialized by manual setting of a seed point within the SC in each slice. Manual corrections of the outlines were performed when necessary. Lesions were segmented entirely manually. This segmentation was performed by two medical students (LH and VP) after an intensive training period supervised by a senior neuroradiologist (JK). While annotating the 4 mm axial slices, the students additionally reviewed both the sagittal images and the primary MR report for comparison. Ambiguous cases were resolved by a senior neuroradiologist. We performed a cross-validation (LH and VP) in 25 patients, 15 of whom had SC lesions, indicating very good agreement (mean voxel-wise Dice coefficient of atrophy/lesions: 0.98/0.79). Additionally, the inferior border of the second cervical vertebra (C2) and the medullary cone were marked separately. These segmentations were evaluated using an in-house python-based algorithm.

We rigidly registered axial and sagittal scans with the SimpleITK software, version 1.20, using six degrees of freedom, to combine the segmentations of the consecutive axial stacks. Registration was initialized using the global coordinate system of the images and based on mattes mutual information with a bin size of 50. To account for missing slices, we used linear interpolation to insinuate the curvature of the missing region and used a nearest neighbour search to determine the thickness of the interpolated regions. These combined masks, covering the entire SC, were subdivided in a region above the C2 landmark and 19 regions between the C2 landmark and the medullary cone, equally divided based on the total stretched length of the skeletonized spine along the cranio-caudal axis (i.e., the spinal centerline). In analogy to the nerve roots, these 19 regions were named C3–C8, T1–T12, and L1. Assuming equal distances between the root entry zones of the SC, which is roughly the case in the upper SC, a region labelled [C or T]x approximately covers the part of the SC between the root entry zones [C or T]x and [C or T]x + 1, while the maximum of the lumbar enlargement corresponds to T11.

### Statistical Analyses

To capture the spatial distribution of the lesion volume along the spine, we computed the mean lesion volume for each segment and each MS type separately. Additionally, we plotted the percentage of detected lesion volume (cranio-caudal direction) against spinal cord levels to visualize the increase in sensitivity with an increasing number of SC levels covered. Furthermore, we computed the ratio of patients with a lesion when only checking the upper segments. Patients with/without lesions are reported as absolute or relative frequencies.

## Results

We identified 575 patients aged between 18 and 60 years with an established diagnosis of CIS or MS and available MRI of the SC. MRI data from 545 patients showed sagittal and axial coverage from at least the inferior border of C2 to the medullary cone; two of these had to be excluded due to poor image quality, leaving 543 MRI data sets for the analysis. The demographics of the resulting cohort are summarized in Table 1.

**Table 1:** Characteristics of the patients

	CIS	RRMS	SPMS	PPMS	All
*N*	37	442	38	26	543
*Females* (in %)	20 (54%)	304 (69%)	22 (58%)	12 (46%)	358 (66%)
*Age* (in years)	34.3 ± 9.1 34.9 [20.1–55.4]	36.1 ± 9.6 35.3 [18.0–59.9]	47.4 ± 6.6 49.5 [33.1–59.6]	47.1 ± 8.5 46.8 [30.8–59.6]	37.3 ± 10.0 36.6 [18.0–59.9]
*Disease duration* (in years)	0.5 ± 0.9 0.08 [0.0–3.4]	4.0 ± 6.1 1.1 [0.0–34.8]	14.3 ± 8.7 13.4 [0.3–32.3]	5.2 ± 3.9 3.9 [0.06–13.3]	4.6 ± 6.7 1.3 [0.0–34.8]
*EDSS*	1.1 ± 0.9 1.0 [0.0–3.5]	1.6 ± 1.4 1.5 [0.0–8.5]	5.6 ± 1.6 6.0 [2.0–9.0]	4.1 ± 1.7 3.5 [1.5–8.5]	2.0 ± 1.8 1.5 [0.0–9.0]
*Patients with SC lesions* (in %)	27%	77%	95%	85%	75%
*Spinal lesion volume* (in ml)	0.04 ± 0.09 0.0 [0.0–0.4]	0.4 ± 0.8 0.1 [0.0–11.6]	1.1 ± 1.7 0.6 [0.0–9.6]	0.6 ± 0.7 0.4 [0.0–3.4]	0.4 ± 0.9 0.1 [0.0–11.6]
*Number of spinal lesions*	0.5 ± 1.0 0 [0–4]	3.2 ± 3.6 2 [0–25]	5.8 ± 4.1 6 [0–17]	4.5 ± 3.7 4 [0–12]	3.3 ± 3.6 2 [0–25]

Values are given in mean ± standard deviation, median and range

*CIS* clinically isolated syndrome, *EDSS* Expanded Disability Status Scale, *PPMS* primary progressive multiple sclerosis,* RRMS* relapsing-remitting multiple sclerosis,* SC* spinal cord, *SPMS* secondary progressive multiple sclerosis

**Fig. 1:**
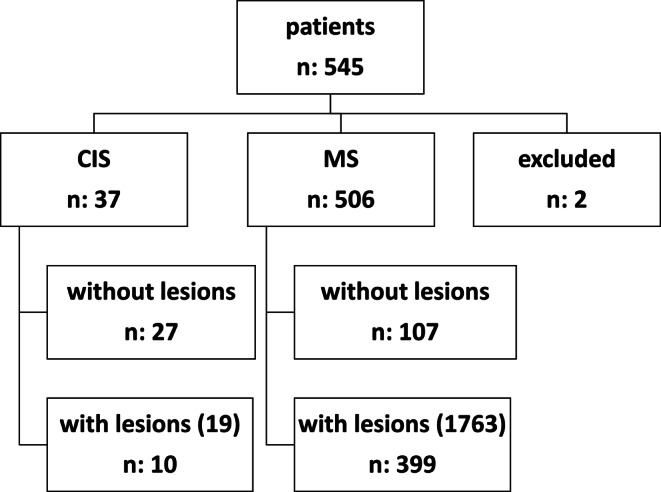
Flowchart showing patients with diagnosis of clinically isolated syndrome (CIS) or multiple sclerosis (MS) and number of patients with lesions. The values given in brackets indicate the numbers of lesions

**Fig. 2:**
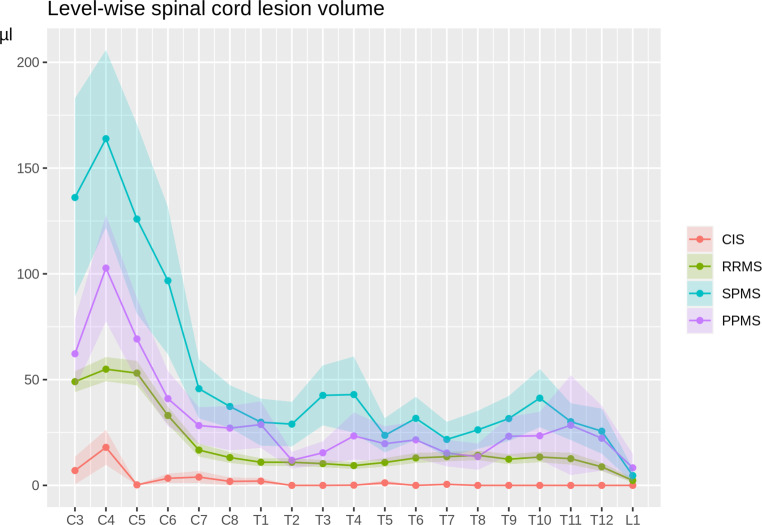
Distribution of spinal lesion volume (in µl) across spinal cord levels. The shaded areas show the standard error of the mean. The level-wise lesion volumes peaked at the level of C4 and showed significant differences for MS-subtypes (CIS < RRMS < PMS) as indicated. *CIS* clinically isolated syndrome (*red curve*), *PPMS* primary progressive multiple sclerosis (*purple curve*), *RRMS* relapsing-remitting multiple sclerosis (*green curve*), *SPMS* secondary progressive multiple sclerosis (*blue curve*)

**Fig. 3:**
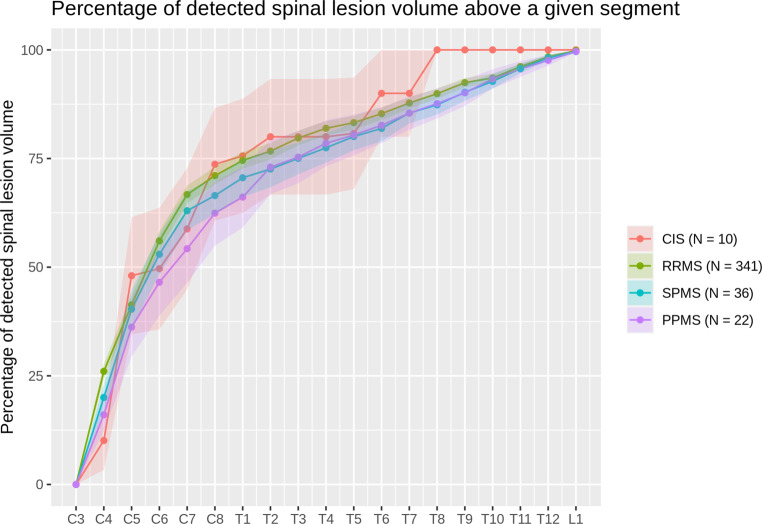
Diagram showing the percentage of detected spinal lesion volume above a given spinal cord segment. The shaded areas show the standard error of the mean. *CIS* clinically isolated syndrome (*red curve*), *PPMS* primary progressive multiple sclerosis (*purple curve*), *RRMS* relapsing-remitting multiple sclerosis (*green curve*), *SPMS* secondary progressive multiple sclerosis (*blue curve*)

**Fig. 4:**
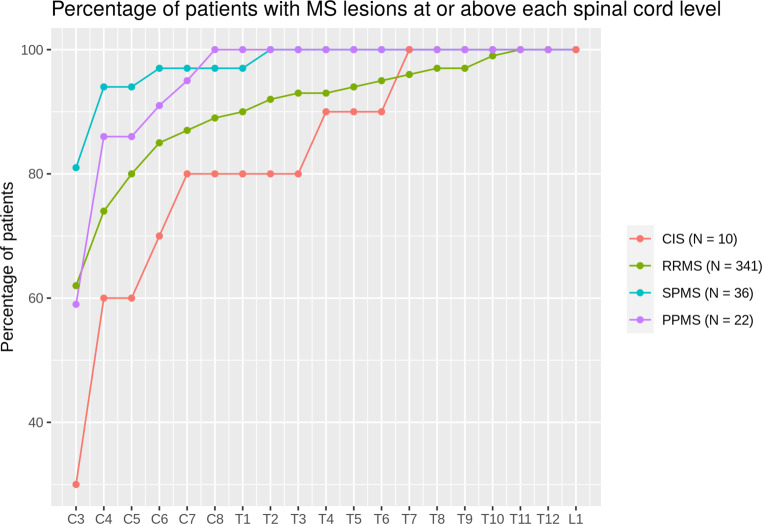
Diagram showing the percentage of detected patients with MS lesions in correlation to the assessment of the different segments of the vertebral bodies. *CIS* clinically isolated syndrome (*red curve*), *PPMS* primary progressive multiple sclerosis (*purple curve*), *RRMS* relapsing-remitting multiple sclerosis (*green curve*), *SPMS* secondary progressive multiple sclerosis (*blue curve*)

A total of 1782 lesions were detected in 409 patients (Fig. 1, 19 lesions in 10 patients with CIS, and 1763 lesions in 399 patients with MS). The level-wise lesion volumes peaked at the level of C4 and showed significant differences for MS-subtypes (CIS < RRMS < PMS) as indicated in Fig. 2. The percentage of detected spinal lesion volume above a given spinal cord segment is illustrated in Fig. 3. Figure 4 shows the percentage of patients with MS lesions detected in relation to the assessment of the different vertebral segments.

As the FoV of an isolated examination of the cervical SC mostly included the upper part of the thoracic spine up to the 3rd thoracic vertebral body, following descriptions refer to this level. 70% of the lesion volume was located above the level of the 3rd thoracic vertebral body. 26 patients (6%) (MS, *n*: 24 (6%); CIS, *n*: 2 (20%)) had lesions exclusively below the level of the 3rd thoracic vertebral body, i.e., 94% of all patients with spinal lesions were detected when MRI scans of the upper part of the SC were evaluated exclusively. Differences in the percentage of detected patients with MS lesions between groups are best explained by differences in the overall frequency of lesions rather than by differences in lesion distribution, which was very similar in all patient groups (Fig. 2).

## Discussion

The results of our study confirm that most lesions are located in the upper segment of the SC down to the level of the 3rd thoracic vertebral body, an area that represents the FoV of an isolated MRI of the cervical SC as routinely performed in many centers. However, SC lesions occurred at all SC levels down to the medullary cone.

Our study is in agreement with a previous study [12] which showed that the majority of MS lesions were located in the upper part of the SC. However, it was shown that approximately 20% of the lesions occurred in the lower part of the SC in MS patients [12] and in 41% of patients with MOG-AB associated diseases [13]. Therefore, it is essential to scan the whole SC in order to be able to exclude differential diagnosis such as MOG-AB. In addition, a small number (8%) of MS patients had lesions exclusively below the level of the 5th thoracic vertebra [12]. In contrast to this study, we included a larger cohort of patients and used a different semi-automated segmentation tool.

Another unique feature of our study is that we analyzed and segmented the lesion volume on axial images. It might be difficult to detect lesions in lateral localization with sagittal images, which are more prone to partial volume artifacts. Other studies have analyzed lesions primarily on sagittal images, sometimes in combination with selected axial slices [1, 14–16]. In addition, we analyzed lesions down to the conus medullaris, thus covering the entire SC in a representatively large cohort of patients. We are aware of only a few studies performing whole-spine MRI analysis in MS patients [12, 16]. In contrast to a recent article with only 74 patients [16], we analyzed considerably more patients (*n*: 543). Furthermore, we performed axial imaging of the entire SC and not only at the level of the lesions found on sagittal images. We also analyzed lesion volume.

It is important to emphasize that the clinical context of MRI examinations might be very different. SC imaging can be essential to fulfill the criteria of dissemination in space and thus, might have an impact on the right and early diagnosis and startpoint of treatment. On the other hand, SC imaging might show the extend of disease activity and then could turn to changes in patient management.

As recently published, SC lesions are of high prognostic value because patients with SC lesions are more likely to suffer from progression independent of relapse activity [17]. Secondary progressive MS (SPMS) is a form of MS that is characterized by the progression of disability following a relapsing-remitting form of MS (RRMS). Patients with SPMS have a higher SC lesion load and SC atrophy than patients with RRMS [18]. As an early onset of a high SC lesion load is a good predictor of conversion from RRMS to SPMS and an indicator for the degree of future disability total SC MRI in MS patients in early stages could therefore be useful for treatment decisions, such as the prescription of disease-modifying drugs. SC lesion number and SC lesion volume could be added to a prognostic algorithm to support treatment decision making [19]. Further prospective and multicenter studies are needed for validation, such as the planned study based on the ProVal-MS cohort (ProVal-MS study; German Clinical Trails Register study ID: DRKS00014034).

A limitation of our study was the difficulty to count confluent lesions separately or to detect diffuse lesions. However, these types of lesions are more common in patients with a higher disease burden such as PPMS or SPMS, and these patient groups represent only a small part of the patient cohort included in our study. Another limitation is that the results may be influenced by false-negative detections which are likely to differ across segment levels. In cervical levels false-positive lesions might be produced be swallowing artifacts whereas in thoracal levels pulsation artifacts of the cardiovascular system might occur. However, we performed a cross-validation in 25 patients with a very good agreement and ambiguous cases were resolved by a senior neuroradiologist. In addition, volumetric calculations using 2D data acquired at 4 mm slice thickness are prone to partial volume artifacts and may be less accurate than 3D data acquired at isotropic spatial resolution, which was not available in this study. Finally, we evaluated only T2w images, but guidelines [20] recommend at least two different sagittal sequences (T2w, PD, or STIR) to improve sensitivity.

In conclusion, the FoV of an isolated MRI acquisition of the cervical SC (up to the level of the 3rd thoracic vertebra) is sufficient to detect 94% of all patients with CIS or MS and SC lesions. However, a small percentage (6%) had only lesions below this level. Therefore, we recommend examining the whole SC at least in cases of unclear diagnoses or first diagnoses of CIS and MS in the clinical setting.

## Abbreviations

CIS: Clinically isolated syndrome
EDSS: Expanded Disability Status Scale
FoV: field of view
MOG-AB: myelon oligodendrocyte glycoprotein antibody
MRI: magnetic resonance imaging
MS: multiple sclerosis
PPMS: primary progressive multiple sclerosis
RRMS: relapsing-remitting multiple sclerosis
SPMS: secondary progressive multiple sclerosis
SC: spinal cord
STIR: short tau inversion recovery
TSE: turbo spin echo
